# Nephroprotective Efficacy of Sugammadex in Ischemia-Reperfusion Injury: An Experimental Study in a Rat Model

**DOI:** 10.7759/cureus.15726

**Published:** 2021-06-17

**Authors:** Mehmet Tercan, Ferda Yılmaz İnal, Hatice Seneldir, Hasan Kocoglu

**Affiliations:** 1 Department of Anesthesiology and Reanimation, University of Health Sciences, Mehmet Akif Inan Research and Training Hospital, Sanliurfa, TUR; 2 Department of Anesthesiology and Reanimation, Istanbul Medeniyet University, Istanbul, TUR; 3 Department of Medical Pathology, Istanbul Medeniyet University, Istanbul, TUR

**Keywords:** rat models, kidney injury, preconditioning, sugammadex, ischemia-reperfusion

## Abstract

Background: It is known that ischemia-reperfusion damage in the kidney is one of the most common causes of acute kidney failure. It is also known that reduced renal damage has a nephroprotective effect by reducing the release of inflammatory and vasoactive peptides that cause tissue damage. Therefore, we think that reperfusion caused by ischemia in kidney damage may be an important focus for clinical research.

Methods: A total of 21 healthy 230-250 g female rats were used in our experimental study. During the experiment, animals were randomly divided into three groups, each containing seven rats. Group 1: The group that underwent left nephrectomy with a sham operation. Group 2: Left renal ischemia for 60 minutes, then left nephrectomy followed by 45 minutes of reperfusion. Group 3: Left renal ischemia for 60 minutes, then reperfusion for 45 minutes, followed by left nephrectomy. In this group, sugammadex was given intravenously at a dose of 100 mg/kg at the beginning of reperfusion. In the histomorphological examination, damage findings of tubules atrophy, dilation and cast formation, tubular epithelial brush border loss and vacuolization, presence of fibrosis as interstitial structural change, capillary vasodilatation/congestion and neutrophilic cell infiltrates in interstitial spaces, and morphological changes in glomeruli were evaluated.

Results: When evaluated based on tubular brush border, there were no significant differences between Group 2 and Group 1 (P = 0.454), while the damage in Group 3 was less significant than Group 2 (P = 0.017). When evaluated in terms of tubular vacuolization, there was no significant difference between Group 2 and Group 1 (P = 0.902), while the damage in Group 3 was less significant than Group 2 (P = 0.017).

Conclusion: We believe that 100 mg/kg sugammadex given at the beginning of reperfusion after one hour of ischemic condition on rats has a histochemically detectable nephroprotective effect.

## Introduction

Resumption of blood flow to an organ after a critical period of ischemia causes parenchymal injury. This phenomenon occurring through different clinical and laboratory findings is generally known as ischemia-reperfusion (IR) injury [1]. Histologically, it has been shown that acute tubular necrosis develops during/after renal IR injury [2]. Organ dysfunction accompanying this condition is generally associated with increased microvascular permeability, interstitial edema, impaired vasoregulation, inflammatory cell infiltration, and parenchymal cell dysfunction and necrosis [3]. Ischemic injuries to vital organs such as the heart, brain, and kidneys can contribute to increased morbidity and mortality. Carotid endarterectomy, kidney, and liver transplantation operations are common procedures, and reperfusion develops following the ischemia period in the tissues. Partial nephrectomy operations frequently used in urological surgery are a good example of controlled IR applications. If IR damage develops as a result of the controlled ischemia applied in such operations, it will reduce the already reduced functional kidney units due to surgery, and a situation may arise for both patients and clinicians. It is known that IR damage in the kidney is one of the most common causes of acute renal failure [4,5]. It is also acknowledged that reduced renal damage has a nephroprotective effect by reducing the release of inflammatory and vasoactive peptides that cause tissue damage [6]. Therefore, we think that reperfusion caused by ischemia in kidney injury may be an important focus for clinical research.

Sugammadex is a modified ɣ-cyclodextrin molecule that has recently been used widely in daily anesthesia applications. Neuroprotective effects of cyclodextrins have been shown in various previous studies [7,8]. Our research did not find an article on the preconditioning effect of sugammadex on renal IR damage. This experimental study aims to determine the preconditioning role of sugammadex on histopathological changes caused by renal IR injury in rats by creating a precondition model with IR and sugammadex.

## Materials and methods

This study was initiated after the approval of the Bezmialem Vakif University Animal Experiments Local Ethics Committee (Protocol number: 21.02.2020-2020/30). In our experimental study, a total of 21 healthy female, two- to three-month-old 230-250 g Sprague-Dawley rats were used.

Animals and experimental protocol

During the experiment, these rats were kept alive in rooms with light/dark lighting for 12-hour periods and automatically adjusted for temperature (22 ± 2ºC) and humidity (60 ± 5%). Before starting the experiment, the animals were adapted to the ambient conditions for a week. During this time, all rats were allowed free access to standard rat food and water in their cages. Animals were fasted overnight before experiments but were given free access to water. Intramuscular anesthesia was applied to all subjects with 50 mg/kg ketamine hydrochloride (HCl) (Ketalar® Vial, Eczacibasi Pharmaceutical Co., Istanbul, Turkey) and 20 mg/kg xylazine HCl (Rhompon® Vial, Bayer, Istanbul, Turkey).

During the experiment, the animals were randomly divided into three groups of seven rats each:

Group 1: Sham control group (n = 7). The group whose abdomen was dissected by performing a sham operation, but left nephrectomy was performed without clamping the kidney.

Group 2: IR (n = 7). Group in which the abdomen was dissected and given left renal ischemia for 60 minutes, then left nephrectomy after 45 minutes of reperfusion.

Group 3: IR + sugammadex-treated group (n = 7). Group in which the abdomen was dissected and given left renal ischemia for 60 minutes, then left nephrectomy after 45 minutes of reperfusion. In this group, sugammadex (Bridion 200 mg/2 mL vial, MSD, USA) was administered intravenously at a dose of 100 mg/kg at the beginning of reperfusion.

After left lateral laparotomy, the left renal artery and vein were clamped for 60 minutes with a hemostasis clip to create the IR model. The clip was then removed to allow reperfusion. After nephrectomy, rats were sacrificed by creating diaphragm rupture. This was determined as the endpoint of the experiment.

Histopathological evaluation

The dissected nephrectomy specimens were fixed in 10% neutral formaldehyde solution for 24 hours. On macroscopic examination, the kidneys were cut from the lateral surface to the hilar area in two halves, one being anterior and posterior. Sagittal sections of 3-mm thickness containing the cortex and medulla were taken from each specimen. Tissue samples were embedded in paraffin blocks after routine tissue monitoring (Leica TP1020, Nussloch, Germany). The 4-μm-thick sections prepared from the paraffin blocks in the microtome were stained with hematoxylin-eosin (H&E) dye in an automatic stainer. All sections of the preparations were examined histomorphologically under an Olympus BX51 fluorescence microscope (Olympus, Tokyo, Japan) by a single pathologist blindly.

In the histomorphological examination, atrophy in the tubules, dilatation and cast formation, brush-like edge loss and vacuolization in the tubulus epithelium, presence of fibrosis as interstitial structural changes, capillary vasodilation/congestion in interstitial areas and neutrophilic cell infiltration, and morphological changes in the glomeruli were evaluated. Histological damage scoring was semiquantitatively graded from 0 to 4 as 0 = none, 1 = 1%-25%, 2 = 26%-50%, 3 = 51%-75%, and 4 = 76%-100%.

Statistical analysis

All data were analyzed using Statistical Package for Social Sciences (SPSS) version 20 (IBM Corp., Armonk, NY, USA). Differences between data were evaluated using the Mann-Whitney U test. Statistical significance level was accepted as P < 0.05.

## Results

A total of 21 Wistar rats were included in the study, in three equal groups. The parameters evaluated in the histological classification and the data of the rats are given in Table 1.

**Table 1:** Parameters evaluated in histological classification

	Neutrophilic cell infiltration	Capillary vasodilation/congestion	Tubular atrophy	Tubular brush border loss	Tubular dilatation	Cast formation	Interstitial structural changes	Renal corpuscler morphology	Tubular vacuolization
Group 1 Rat 1	0	2	0	0	0	1	0	3	2
Group 1 Rat 2	0	1	0	2	0	0	0	0	1
Group 1 Rat 3	0	2	0	1	1	1	0	0	1
Group 1 Rat 4	0	0	0	1	1	0	0	0	2
Group 1 Rat 5	0	0	0	1	0	0	0	0	1
Group 1 Rat 6	0	2	0	1	0	0	0	3	1
Group 1 Rat 7	0	1	0	2	0	0	0	0	2
Group 2 Rat 1	0	3	0	1	0	0	0	0	1
Group 2 Rat 2	0	1	0	1	0	0	0	0	1
Group 2 Rat 3	0	4	0	2	1	0	0	3	2
Group 2 Rat 4	0	0	0	1	1	0	0	0	1
Group 2 Rat 5	0	4	0	3	0	0	0	3	3
Group 2 Rat 6	0	1	0	1	1	1	0	0	1
Group 2 Rat 7	0	4	0	2	1	0	0	3	2
Group 3 Rat 1	0	1	0	0	0	0	0	0	0
Group 3 Rat 2	0	1	0	0	0	1	0	0	0
Group 3 Rat 3	0	1	0	0	0	0	0	0	0
Group 3 Rat 4	0	3	0	1	0	1	0	1	1
Group 3 Rat 5	0	2	0	1	0	0	0	0	1
Group 3 Rat 6	0	4	0	1	0	0	0	1	1
Group 3 Rat 7	0	0	0	0	0	0	0	0	0

The data of the total score evaluations of the three groups are given in Table 2. In the analysis performed, there was no significant difference between the groups in terms of total score evaluations (P > 0.05).

**Table 2:** Total score evaluations

Groups	Point
Group 1 (n = 7)	4 (2-8)
Group 2 (n = 7)	5 (3-13)
Group 3 (n = 7)	2 (0-7)

Parameters showing the acute histological damage of the groups are given in Table 3. When evaluated in terms of capillary congestion, there was no difference between Group 2 and Group 3 (P = 0.073) and Group 2 and Group 1 (P = 0.456). While there was no significant difference between Group 2 and Group 1 (P = 0.454) when evaluated in terms of tubular brush border, the damage in Group 3 was significantly less than that in Group 2 (P = 0.017). When compared in terms of renal corpuscular morphology, there was no significant difference between Group 2 and Group 3 (P = 0.456) and Group 2 and Group 1 (P = 0.710). While there was no significant difference between Group 2 and Group 1 (P = 0.902) when evaluated in terms of tubular vacuolization, the damage in Group 3 was significantly less than that in Group 2 (P = 0.017).

**Table 3:** Parameters showing the histological damage status ^a^p = 0.456 compared with Group 2 to Group 3; ^b^p = 0.209 compared with Group 2 to Group 1; ^c^p = 0.017 compared with Group 2 to Group 3; ^d^p = 0.454 compared with Group 2 to Group 1; ^e^p = 0.456 compared with Group 2 to Group 3; ^f^p = 0.710 compared with Group 2 to Group 1; ^g^p = 0.017 compared with Group 2 to Group 3.

Groups	Capillary vasodilation/congestion	Tubular brush border loss	Renal corpuscular morphology	Tubular vacuolization
Group 1 (n = 7)	2 (0-2)^b^	1 (0-2)^d^	0 (0-3)^f^	1 (1-2)^h^
Group 2 (n = 7)	4 (0-4)	1 (1-3)	0 (0-3)	1 (1-3)
Group 3 (n = 7)	1 (0-4)^a^	0 (0-1)^c^	0 (0-1)^e^	0 (0-1)^g^

**Figure 1:**
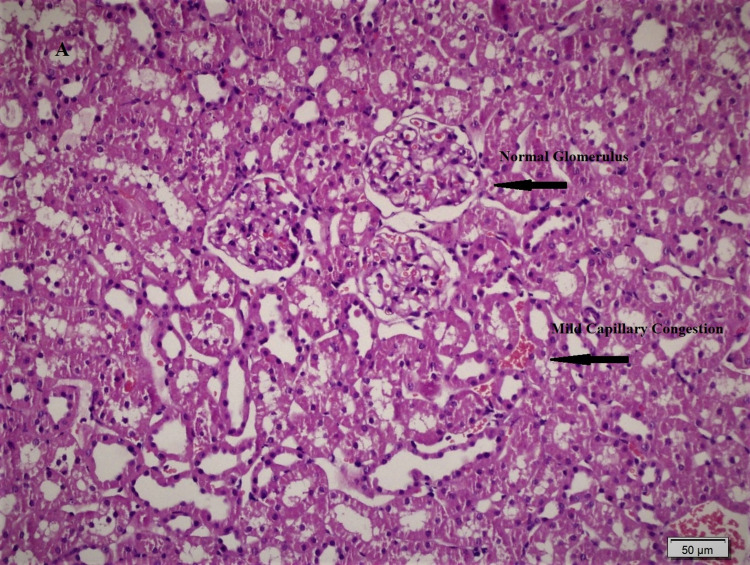
The sham-operated group showed mild degree capillary congestion in the renal interstitium (H&Ex200).

**Figure 2:**
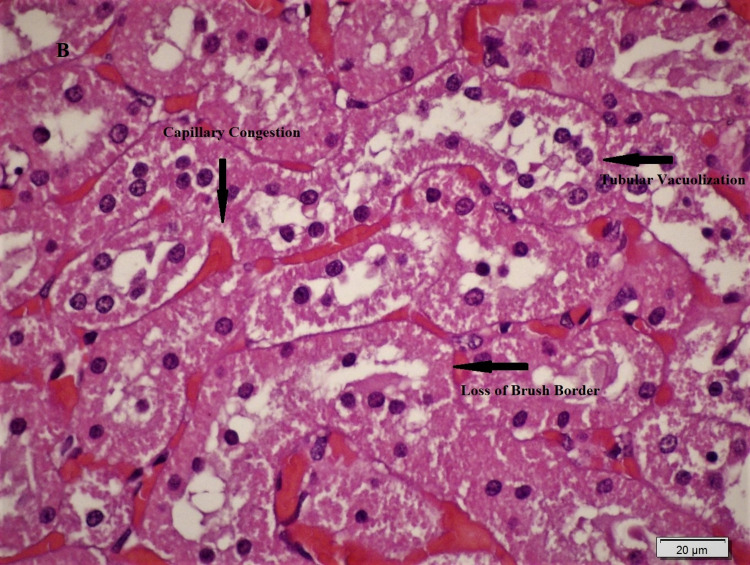
Kidneys of untreated ischemia rats showed loss of brush border and vacuolization in tubular epithelial cells and moderate-severe degree capillary congestion in the renal interstitium (H&Ex600).

**Figure 3:**
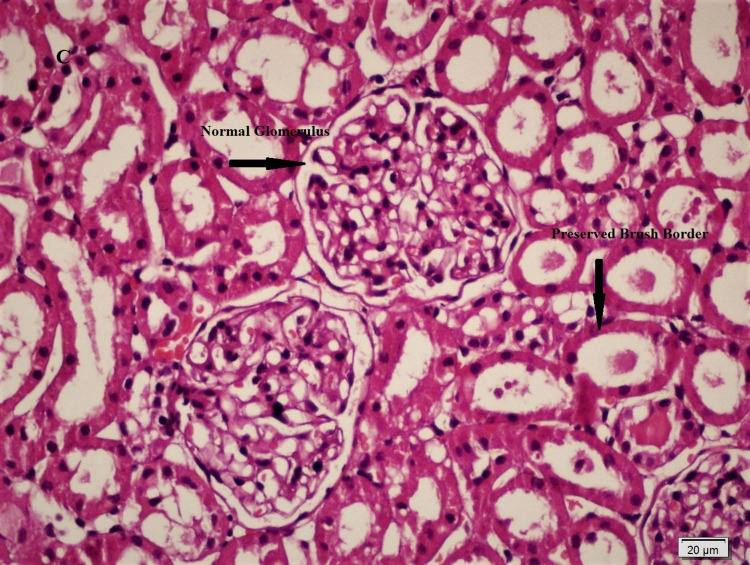
The group of kidneys treated by injecting sugammadex showed normal glomeruli and preserved brush border in the tubular epithelial cells (H&Ex400).

Histopathological differences between the groups are shown in Figures 1, 2, 3, and significant differences were detected between Group 2 and Group 3 in ischemic damage markers.

## Discussion

In the IR model created with this experimental study on rats, we found findings that sugammadex may play a protective role in kidney damage. Renal ischemia that develops during arterial occlusion, shock, and organ transplantation is generally associated with cell death, renal failure, delayed graft function in kidney transplantation, and renal graft rejection [9]. Following an acute renal ischemia attack, early reperfusion continues to be the first-line strategy to limit damage to the kidney. However, renal reperfusion has the potential to cause cellular death, similar to that observed in the heart [10,11]. Therefore, investigation of preventive strategies used during reperfusion is essential to prevent such lesions [12]. For example, urologists have studied various surgical methods in order not to be affected by or reduce the effect of ischemic damage in partial nephrectomy applied in renal carcinomas. Some of these include creating hypothermia in the kidney during the procedure, performing partial nephrectomy without using a clamp or using a laser in dissection [6]. Irreversible ischemic damage can be minimized by pharmacological intervention or surgical approaches such as hypothermia, limited warm ischemia, or zero or segmental ischemia [13].

Ischemic preconditioning is also a method to protect organs from damage that may be caused by ischemia. In our study, we found that sugammadex, which we applied at the beginning of reperfusion after ischemia, decreased kidney damage by causing a decrease in tubular brush border and tubular vacuolization histologically. The effects of different drugs were investigated on kidney IR models created in rats. Kocoglu et al. investigated renal damage and the effects of dexmedetomidine in the IR model they created on 30 rats. In the results of this study, they reported that kidneys of untreated ischemic rats showed tubular cell swelling, cellular vacuolization, pycnotic nuclei, medullary congestion, and moderate to severe necrosis, and kidneys in the dexmedetomidine-injected group showed mild edema in normal glomeruli and tubular cells [14]. This is a rare study in which dexmedetomidine reduces kidney damage caused by IR.

In the study conducted by Kianian et al., 30 mg/kg celecoxib was administered orally to subjects before ischemia. In this study, the gender difference was also investigated, and they reported that celecoxib improves kidney function and histopathological damages and reduces oxidative stress equally in both sexes in similar pathological conditions. In addition, as a result of this study, they stated that these protective effects of celecoxib on IR-induced kidney damage were independent of gender [15]. In an experimental study, early and late tissue preserving effects of hydrogen gas inhalation on IR damage in the kidney were investigated. In this model, 30 rats were used, and 2% hydrogen gas was given to the study group during ischemia before and for one hour after ischemia. The investigators reported that hydrogen gas significantly reduced IR damage in the kidney in the early and late periods in the histopathological examination of nephrectomy material performed after six hours (early stage) and 14 days (late period) of ischemia [16]. The nephroprotective effect of remifentanil and dexmedetomidine was investigated in the IR model created in the kidney. A 30-minute ischemia model and a 30-minute reperfusion model were applied at the end of the experiment. At the end of the experiment, kidney damage and the nephroprotective effects of the drugs given to the study groups were examined by biochemical and histochemical analysis. As a result of this study, they stated that dexmedetomidine may have a protective effect on renal IR damage, but there is no such evidence for remifentanil [17]. In a recently published study, it has been reported that cyclosporine A given 15 mg/kg intraperitoneally does not prevent the harmful effects of IR damage in rat kidneys [18].

The IR model has also been studied on other vital organs besides the kidney, and the tissue-protective effects of both the model and the drugs used still remain a mystery in many subjects. For example, the neuroprotective efficacy of sugammadex has been reported in an experimental model in which global cerebral ischemia was created by bilateral carotid artery clamping [19]. In another study, the effects of dexmedetomidine on myocardial damage and arrhythmia in the cardiac ischemia-reperfusion model were investigated, in which it was stated that the drug was associated with reduced infarction size in regional ischemia and IR damage but had no effect on the incidence of arrhythmia [20]. Broader studies on these organs and the drugs used may reveal the effectiveness of IR-induced tissue damage and protective strategies. When the sources are examined, it will be seen that kidney IR models differ. The effects of ischemic preconditioning and postconditioning methods were investigated in a study conducted on 40 rats using the renal IR injury model. They concluded that applying these strategies together or separately could not preserve kidney function after acute IR kidney injury or have a protective effect against tubular cell damage [1]. Oliveira et al., in their study, stated that three-cycle 10-minute ischemic preconditioning showed a superior nephroprotective effect than tramadol administration [21]. In our study, after 60 minutes of renal ischemia, 45 minutes of reperfusion was applied, and possible nephroprotective effects were investigated by histochemical analysis by administering sugammadex in the study group.

Neuromuscular blocking agents have been used in anesthesia for a long time. Various drugs such as neostigmine, suramin, purified human plasma cholinesterase, and cysteine ​​have been used to reverse the effects of these drugs [22]. Cyclodextrins have an important place in our daily life as components of food products, cosmetics, textiles, and various medical products. The common property of all cyclodextrins is that they are water-soluble [23]. Sugammadex is a modified form of gamma-cyclodextrin. Its action is to form water-soluble complexes with steroid neuromuscular blockers at a ratio of 1:1 and rapidly extract the drug from the neuromuscular junction into the plasma. Among the group of neuromuscular blocking agents, it is most selective for rocuronium. Efficacy varies depending on the dose [22]. Sugammadex, which is widely used in anesthesia practice, may cause tissue damage and tissue loss when administered intra-arterially, so patients should be carefully controlled to avoid accidental injection if arterial cannulation is performed [24].

Some limitations of our study should be noted. Most importantly, the detection of renal damage worked only histochemically. Analysis of these findings together with biochemical examinations could provide more concrete evidence. Different results could be obtained with different doses. Necrotic and apoptotic cell evaluation requires special dyes such as tunnel, e-nos, and quantitative counting. We could not look at these parameters in our study because it was not in our laboratory facilities.

## Conclusions

As a result, strategies exist that attempt to prevent or at least reduce the severity of renal injury and damage to renal tubular cells after IR injury. We believe that 100 mg/kg sugammadex given at the beginning of reperfusion after one hour of ischemic state in rats has a histopathologically detectable nephroprotective effect. We believe that large-based studies with frequently used drugs and different strategies in anesthesia practice will be useful in preventing renal damage after ischemia.

## References

[1] Arantes VM, Bueno RT, Módolo RP (2018). Effects of ischemic preconditioning and postconditioning in a renal ischemia-reperfusion injury model: a comparative experimental study in rats. Transplant Proc.

[2] Brezis M, Rosen S (1995). Hypoxia of the renal medulla--its implications for disease. N Engl J Med.

[3] Granger DN, Korthuis RJ (1995). Physiologic mechanisms of postischemic tissue injury. Annu Rev Physiol.

[4] Moens AL, Claeys MJ, Timmermans JP, Vrints CJ (2005). Myocardial ischemia/reperfusion-injury, a clinical view on a complex pathophysiological process. Int J Cardiol.

[5] Bellomo R, Kellum JA, Ronco C (2012). Acute kidney injury. Lancet.

[6] Malthouse T, Kasivisvanathan V, Raison N, Lam W, Challacombe B (2016). The future of partial nephrectomy. Int J Surg.

[7] Abulrob A, Tauskela JS, Mealing G, Brunette E, Faid K, Stanimirovic D (2005). Protection by cholesterol-extracting cyclodextrins: a role for N-methyl-D-aspartate receptor redistribution. J Neurochem.

[8] Frank C, Rufini S, Tancredi V, Forcina R, Grossi D, D'Arcangelo G (2008). Cholesterol depletion inhibits synaptic transmission and synaptic plasticity in rat hippocampus. Exp Neurol.

[9] Perico N, Cattaneo D, Sayegh MH, Remuzzi G (2004). Delayed graft function in kidney transplantation. Lancet.

[10] Sharfuddin AA, Molitoris BA (2011). Pathophysiology of ischemic acute kidney injury. Nat Rev Nephrol.

[11] Verma S, Fedak PW, Weisel RD (2002). Fundamentals of reperfusion injury for the clinical cardiologist. Circulation.

[12] Li J, Iorga A, Sharma S (2012). Intralipid, a clinically safe compound, protects the heart against ischemia-reperfusion injury more efficiently than cyclosporine-A. Anesthesiology.

[13] Mir MC, Ercole C, Takagi T (2015). Decline in renal function after partial nephrectomy: etiology and prevention. J Urol.

[14] Kocoglu H, Ozturk H, Ozturk H, Yilmaz F, Gulcu N (2009). Effect of dexmedetomidine on ischemia-reperfusion injury in rat kidney: a histopathologic study. Ren Fail.

[15] Kianian F, Seifi B, Kadkhodaee M, Sajedizadeh A, Ahghari P (2019). Protective effects of celecoxib on ischemia reperfusion-induced acute kidney injury: comparing between male and female rats. Iran J Basic Med Sci.

[16] Akdeniz E, Bostancı Y, Özden E (2013). Inhaled hydrogen gas therapy for prevention of renal ischemia/reperfusion injury in rat model. Turkiye Klinikleri J Med Sci.

[17] Erkılıç E, Kesimci E, Alaybeyoğlu F (2017). Does remifentanil attenuate renal ischemia-reperfusion injury better than dexmedetomidine in rat kidney?. Drug Des Devel Ther.

[18] Oliveira ACC, Módolo NSP, Domingues MAC, Schwingel PA (2019). Effects of cyclosporine on ischemia-reperfusion injuries in rat kidneys. An experimental model. Acta Cir Bras.

[19] Ozbilgin S, Yılmaz O, Ergur BU (2016). Effectiveness of sugammadex for cerebral ischemia/reperfusion injury. Kaohsiung J Med Sci.

[20] Kocoglu H, Karaaslan K, Gonca E, Bozdogan O, Gulcu N (2008). Preconditioning effects of dexmedetomidine on myocardial ischemia/reperfusion injury in rats. Curr Ther Res Clin Exp.

[21] Oliveira RC, Brito MV, Ribeiro RF Júnior (2017). Influence of remote ischemic conditioning and tramadol hydrochloride on oxidative stress in kidney ischemia/reperfusion injury in rats. Acta Cir Bras.

[22] Naguib M (2007). Sugammadex: another milestone in clinical neuromuscular pharmacology. Anesth Analg.

[23] Kurkov SV, Loftsson T (2013). Cyclodextrins. Int J Pharm.

[24] Kiraz HA, Toman H, Erbas M, Yuyucu Karabulut Y, Simsek T, Hanci V, Uzun M (2016). Investigation of histopathological effects after intra-arterial sugammadex injection in an experimental animal model. Bratisl Lek Listy.

